# Show yourself, a short film to show professionals at an admission ward your ‘euthymic being’ during an admission for mania

**DOI:** 10.1186/s40345-018-0136-6

**Published:** 2019-01-04

**Authors:** P. J. J. Goossens, T. H. Daggenvoorde, H. G. Groot Lipman, S. Verhaeghe, A. W. M. M. Stevens

**Affiliations:** 1 SCBS Bipolar disorders, Dimence group, Deventer, The Netherlands; 2 University Centre for Nursing and Midwifery, Department of Public Health, Faculty of Medicine and Health Sciences, Ghent University, Ghent, Belgium; 3 Radboud Institute for Health Sciences IQ Healthcare, Radboud University Medical Center, Nijmegen, The Netherlands; 4 Gelre Hospital, Apeldoorn, The Netherlands; 5 VIVES Hogeschool, Campus Roeselare, Roeselare, Belgium; 6 Department of Psychiatry, VU University Medical Center, Amsterdam, The Netherlands

**Keywords:** Bipolar disorder, Mania, Self-management, Film

## Abstract

**Background:**

The progress and recovery of a patient with mania during hospitalization is differently seen by professionals working at an admission ward and by relatives of the patient. Professionals often indicate that the situation of the patient is improving while relatives estimate the improvement to be minimal in relation to the recovery of the patient.

**Objective(s):**

To develop an intervention to give professionals at an admission ward an impression of the patient in a euthymic mood state to provide professionals with information to plan and conduct individualized patient centred care.

**Methods:**

Professionals, patients, and relatives were individually interviewed about the preferable content and use of a film in which patients’ shows their ‘euthymic being’. Content analysis was performed.

**Results:**

An outline for the content and use of the film was developed.

**Conclusions:**

The intervention holds promise for clinical practice, but further development and testing is necessary.

## Introduction

Despite treatment, the overall recurrence risk in bipolar disorder is between 39.3 and 55.2% (Vázquez et al. 2015). When a recurrence of a manic episode occurs, admission to a hospital is often needed (Kupka et al. 2015). And during hospitalization, the progress and recovery of the patient can be assessed differently by professionals working at the admission ward and by relatives of the patient. In our clinical practice, relatives of patients who were recently admitted expressed their fear that their family member would be discharged from the hospital too soon. They felt burdened in the period prior to admission and finally felt relieved when their family member was admitted. While professionals of the admission ward told that the condition of the patient had improved since the moment of admission, the relatives noticed hardly any improvement. Moreover, many of them felt exhausted since it often takes a long time before the patients’ behaviour is dangerous enough to justify forced hospitalisation (Beentjes et al. 2016). During hospitalization, family members often experience a lack of communication and involvement in the treatment process (Testerink et al. 2018). The difference in view between the relatives and the professionals at the admission ward might be caused by a difference in perspective between relatives and professionals. The perspective of relatives is their family member in a euthymic mood state while the professionals’ perspective is the patients’ condition at the moment of admission. Therefore it is important that nurses know the patients’ daily activities and habits when he is in a euthymic mood state and act in line with it.

For this reason, a project was started with the aim of overcoming this difference in perspective and to enhance a shared vision about the state of the patient. The idea to achieve this was to record a short film of the patient during a euthymic mood state. This film can be stored in the electronic patient record (EPR) or handed over by the patient or the relatives to the staff of the admission ward, when the patient is admitted. In that way, the film can give the staff an impression of the patient when he is stable.

The project was initiated in Dimence, a large mental health organization in the eastern part of the Netherlands. Within this organization 800 patients with bipolar disorder receive treatment at four outpatient clinics by a specialized multidisciplinary team of psychiatrists, psychologists, nurse practitioners and ambulatory care nurses. Treatment is provided in concordance with the Dutch multidisciplinary guideline on bipolar disorders (Kupka et al. 2015). When, due to recurrence, hospitalization is necessary patients are admitted at a psychiatric high intensive care unit.

## Methods

A qualitative survey study was carried out among patients with bipolar disorder who were admitted because of a (recent) manic episode but euthymic at the moment (n = 3), relatives of these patients (n = 2), a psychiatrist (n = 1) and mental health nurses working at an admission ward (n = 2) or at an outpatient clinic (n = 2). Participants were recruited based on their experience and their willingness to participate. Participants were individually interviewed in the outpatient clinic by a trained interviewer. Three structured questions were asked: (1) what are your views and opinions with regard to this idea? (2) what content needs to be included in the film? and (3) how should this film be used in clinical practice? The participants’ responses to these questions were further explored. Interviews were recorded and transcribed verbatim.

In a qualitative content analysis the transcripts of the individual interviews were read and reread. Those text fragments concerned with the three structured questions were then summarized.

All participants gave their informed consent prior to the start of the interview. According to Dutch legislation formal approval by an ethical committee was not needed since no intervention was carried out.

## Results

All participants saw great potential in the idea of making a film of the patient in a stable condition. Patients stated that it would be worthwhile that professionals working at an admission ward could get an idea on the patients functioning and interacting being in a euthymic mood state. Patients said this could be an adequate tool for the staff to get an impression of who the patient really is being not in an episode. All professionals and relatives approved this idea. Professionals working at an admission ward stated it could give them valuable information to plan, conduct and evaluate more individualized patient-centered care. Professionals working at outpatient clinics assumed it could help their patients during a mental health crisis to connect better with the professionals at the admission ward. All assumed this would probably decrease stress for patient and family.

Content of the film: All participants agreed upon one thing: it is the patient only who can decide what will be filmed. The film should take about 10 min. Patients stated it is important that, before the film is recorded, the patient gets support to write a script.

Patients want to show their daily structure and routines and to talk about their work, how they spend their leisure time and things that are important to them. Patients and relatives want to include what is important for treatment in case of admission based on previous experiences and treatment preferences. Professionals want to see how the patients interact with others and want to see a glimpse of the patients’ personal life and character. They want the patients to talk about how they spend their day, their hobbies, their daily routines and their important values.

All participants stated it was important to record the film in the patient’s home. The professionals mentioned this could give them valuable information about how the patient acts and interacts in his/her own environment. Patients told us that their home is the best place to record the film because they feel at ease and they hope the professional can get a good impression about them.

Use of the film: Most patients prefer the film to be stored in their EPR so it is available to professionals at any time. They state that during a crisis the chance that they will hand over the film, stored on a USB stick, is rather limited due to their condition. One patient preferred her family member to hand over the USB stick in case of admission. In the patients’ treatment plan and relapse prevention plan it needs to be mentioned that a film of the patient in a euthymic state is available, preferably with a hyperlink to the location in the EPR so it is easily available. Based on the results of this small study a manual is developed that can support the professional and the patient to prepare the script for the film (Table 1).

**Table 1:** Topics to be discussed in preparation of the script

*Daily routines*
How does a typical day starts and goes on for you? What are your daily activities and routines?
*Work and education*
Tell something about your work. What kind of (voluntary) work do/did you do—and for how long/often? What is your opinion of your job—how does your job make you feel? Of what significance is your (voluntary) work to you?
If relevant, you can also talk about your education or degrees
*Interests*
Show or tell something about topics you are interested in
Talk about the things you do in your leisure time
*Social circle*
Talk about the significant people in your live and the people whom you have frequently see or speak
Consider if significant others, such as friends and relatives are also included in the recording
*Personality*
Describe something that is typical of your character. As what kind of person do you see yourself? How do you conduct yourself with others?
Consider having a trusted friend or relative describing your character on film
*Home environment*
Talk about your home live and family situation
Consider showing a part of your home
Explain what kind of environment makes you feel comfortable and are important for your state of mind and well-being
*In case of admission*
Based on previous experiences; tell your treatment preferences
What subjects should be talked about and which are better to avoid
*The film can also include other topics, such as*
Religion
Medication
Sleep habits
Life-events and traumas
Pets

## Discussion

With this self-management intervention, patients can contribute actively to their treatment and wellbeing. This is in line with recovery-orientated practices such as Wellness Recovery Action Planning (WRAP) (Cook et al. 2012) and the Illness Management and Recovery program (McGuire et al. 2014). A film about yourself in a stable mood state can be an important addition to the individual WRAP or relapse prevention plan (Daggenvoorde et al. 2013). Some questions can be raised. Firstly: we assume that recording a film can give patients a sense of autonomy in dealing with future crisis. Making a documentary about oneself might have an important therapeutic effect on enhancing reflective capacity and self-efficacy and thus contributing to a higher level of quality of life (Abraham et al. 2014). Secondly: we assume that professionals at an admission ward will value the information presented in the film and will use it for planning and conducting person centered care. But what will the availability of this information mean for them professionally? Thirdly: Implementing innovations in clinical practice is often challenging (Proctor et al. 2009). New practices will not be implemented if potential users do not embrace them (Kimberly and Cook 2008). It is still unclear which specific conditions are needed that enhances implementation and use in clinical practice.

Although all participants see potential in the added value of this intervention, it only gets value if implemented and tested in clinical practice. The development of the intervention is the first step. For the second step, twenty films were recorded and an explorative, descriptive qualitative study is ongoing to examine the experiences of patients with recording the film. Gathering experiences of professionals and patients with the use of the film during an admission of the patient can only take place when patients who recorded a film are admitted due to a manic recurrence. However, the overall recurrence rate in patients with bipolar disorder with a manic episode is rather low (Kupka et al. 2007). Therefore a longitudinal multiple case study with a mixed methods design will be carried out to examine the effect of the intervention on patients outcomes and the experiences of all involved. We hypothesize that due to the more individualized patient centered care length of stay on the admission ward and the use of coercion will decrease. Also, patient satisfaction about the period of hospitalization might increase. As stated earlier we assume that recording the film can contribute to the patients’ self-efficacy in dealing with crisis.

During the interviews participants mentioned other application possibilities of using recordings. For example filming oneself during an episode of mania or depression to reflect on it during treatment. These application possibilities were found beyond the scope of this project but too valuable to ignore. Therefore patients and professionals are interviewed in a separate study about other application possibilities.

Although this intervention was developed for patients with bipolar disorder we assume that it is applicable for other patients’ groups who are at risk for recurrence and therefore in need for admissions too.
